# Drug repurposing based on the similarity gene expression signatures to explore for potential indications of quercetin: a case study of multiple sclerosis

**DOI:** 10.3389/fchem.2023.1250043

**Published:** 2023-09-08

**Authors:** Yulong Chen, Mingliang Zhang, Weixia Li, Xiaoyan Wang, Xiaofei Chen, Yali Wu, Hui Zhang, Liuqing Yang, Bing Han, Jinfa Tang

**Affiliations:** ^1^ School of Pharmacy, Henan University of Chinese Medicine, Zhengzhou, China; ^2^ Henan Province Engineering Research Center for Clinical Application, Evaluation and Transformation of Traditional Chinese Medicine, Henan Provincial Key Laboratory for Clinical Pharmacy of Traditional Chinese Medicine, Henan Province Engineering Research Center of Safety Evaluation and Risk Management of Traditional Chinese Medicine, Department of Pharmacy, The First Affiliated Hospital of Henan University of Chinese Medicine, Zhengzhou, China

**Keywords:** natural products, drug repurposing, drug discovery, quercetin, multiple sclerosis, medicinal indications, omics, experimental verification

## Abstract

Quercetin (QR) is a natural flavonol compound widely distributed in the plant kingdom with extensive pharmacological effects. To find the potential clinical indications of QR, 156 differentially expressed genes (DEGs) regulated by QR were obtained from the Gene Expression Omnibus database, and new potential pharmacological effects and clinical indications of QR were repurposed by integrating compounds with similar gene perturbation signatures and associated-disease signatures to QR based on the Connectivity Map and Coexpedia platforms. The results suggested QR has mainly potential therapeutic effects on multiple sclerosis (MS), osteoarthritis, type 2 diabetes mellitus, and acute leukemia. Then, MS was selected for subsequent animal experiments as a representative potential indication, and it found that QR significantly delays the onset time of classical MS model animal mice and ameliorates the inflammatory infiltration and demyelination in the central nervous system. Combined with network pharmacology technology, the therapeutic mechanism of QR on MS was further demonstrated to be related to the inhibition of the expression of inflammatory cytokines (TNF-α, IL-6, IL-1β, IFN-γ, IL-17A, and IL-2) related to TNF-α/TNFR1 signaling pathway. In conclusion, this study expanded the clinical indications of QR and preliminarily confirmed the therapeutic effect and potential mechanism of QR on MS.

## 1 Introduction

Drug repurposing (also called drug repositioning) is a strategy for identifying new uses for approved or investigational drugs that are outside the scope of the original medical indications. Repurposed drugs can reveal new targets and pathways that can be further exploited (Pushpakom et al., 2019; Loscalzo, 2023). The advantage of drug repurposing is that it takes full advantage of the physical and chemical properties of known approved or experimental drugs and well-defined pharmacological or toxicological mechanisms to shorten the time and cost of new drug development and reduce the potential risk of toxic side effects (Rabben et al., 2021). It is worth noting that most preclinical and clinical trial data are currently available through databases such as DrugBank (https://go.drugbank.com/) and ClinicalTrials (https://beta.clinicaltrials.gov/) (Antoszczak et al., 2020). At present, the method of drug repurposing has been successfully applied to developing new drugs and the repurposing of old drugs has achieved a series of successful cases, which has attracted wide attention from academia and pharmaceutical companies (Parvathaneni et al., 2019). Thalidomide, a drug for pregnancy reaction withdrawn from the market due to severe teratogenicity, was found to have a promising therapeutic effect on multiple myeloma after drug repurposing and has been successfully used in the clinical treatment of the disease (Amare et al., 2021).

Moreover, with the continuous reduction of the cost of high-throughput sequencing technology and the iterative accumulation of data related to drug research and development, massive data obtained by bioinformatics technology to find new pharmacological effects and indications of the marketed drugs and reveal their potential therapeutic mechanism, which has become a hot spot in current drug repurposing research (Xia, 2017; Wang F. et al., 2020). Computational drug repurposing can be used for strategic and purposeful repurposing analysis using reference datasets based on diseases or drugs (Subramanian et al., 2017; Xue et al., 2018). Connectivity Map (CMap) is an interference transcriptome database containing about 30,000 small molecule compounds approved or with potential pharmacological activities (Jiang et al., 2021). Some scholars have successfully applied CMap to drug repurposing and found that benzimidazole compounds (flubendazole, mebendazole, and benzimidazole) may be potential candidates for the treatment of glioblastoma multiforme. Moreover, based on cell experiments also proved that this result of drug repurposing is indeed effective (Ren et al., 2022). The CMap-based drug repurposing approach is also widely used to quickly identify potential drugs for Corona Virus Disease 2019 (COVID-19) (Loganathan et al., 2020; Asano et al., 2022). These successful precedents suggest that this method can be applied to more drug repurposing studies that are widely used in clinical practice or withdrawn due to serious adverse reactions.

Quercetin (QR) is a natural flavonoid that widely exists in nature, such as in vegetables, fruits, and medicinal herbs. Its antioxidant, anti-inflammatory, and antiallergic properties have been observed in numerous *in vitro* and animal studies (Zou et al., 2021). Presently, some clinical trials of QR in treating different diseases have been carried out or completed, including rheumatoid arthritis (Javadi et al., 2017), hyperuricemia (Shi and Williamson, 2016), COVID-19 (ongoing clinical trial ID: QUERCOV), chronic obstructive pulmonary disease (ongoing clinical trial ID: 25738), Alzheimer’s disease (ongoing clinical trial ID: Pro00053594) and type 2 diabetes (completed clinical trial ID: 03-DK-0256). It indicates that the pharmacological effect of QR may be far beyond its existence in food. Therefore, exploring the potential pharmacological effects and clinical indications of QR is still necessary.

**FIGURE 1:**
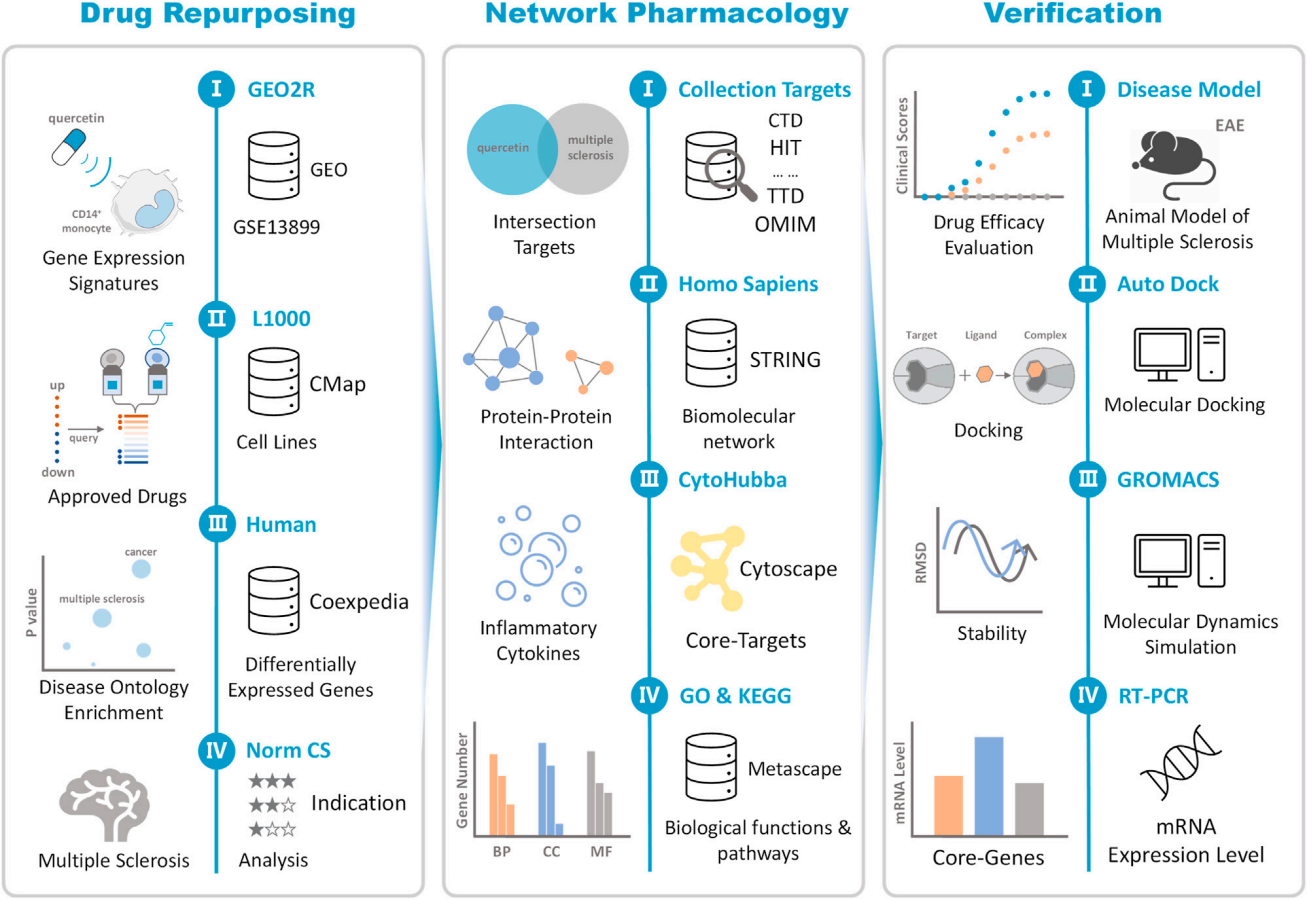
The schematic view of data construction and process. Drug repurposing: Integrating GEO, CMap, and Coexpedia databases, multiple sclerosis (MS) was repositioned as the most relevant potential indication for quercetin (QR). Network pharmacology: Using different databases such as CTD, HIT, TTD, STRING, QR targets, and MS-related molecules are jointly mapped to a biomolecular network. Based on the biomolecular network, the association mechanism between QR and MS is established, and the “network-target-system regulation” mechanism of QR is analyzed. Experimental verification: First, the efficacy of QR was evaluated based on the classic experimental autoimmune encephalomyelitis (EAE) model. In addition, the interaction between QR and critical molecules in the signaling pathway was verified based on molecular docking and molecular dynamics simulation computer technology. Finally, based on RT-PCR to further verify the level of QR regulation of MS-related core molecules.

In this study, differentially expressed genes (DEGs) significantly regulated by QR were obtained from Gene Expression Omnibus (GEO) database. The new potential pharmacological effects and clinical indications of QR were repurposed by integrating clinically approved drugs with similar gene expression signatures to QR and candidate diseases associated with QR-regulated DEGs based on CMap and Coexpedia platform. The classic animal model was used to verify the drug repurposing result and explore the mechanism of action (Figure 1).

## 2 Materials and methods

### 2.1 Drug repurposing

Based on the GEO database (https://www.ncbi.nlm.nih.gov/geo/), the data information on gene expression related to QR regulation was retrieved under the restriction of “*homo sapiens*,” and the GEO online analysis tool GEO2R (https://www.ncbi.nlm.nih.gov/geo/geo2r) was used for data preprocessing and the DEGs in the dataset were screened by the statistical cut-offs of *p*-value <0.05 and |log2-fold change| (|*logFC*|) > 1. Based on CMap (https://clue.io/query) and Drugbank 83 (https://go.drugbank.com/), the approved drugs with the same transcriptional regulation direction as 84 the QR-regulated DEGs were screened out [Normalized Connection Score (Norm CS) ≥ 1.3], and the mechanisms of action between different drugs were compared. Disease Ontology (DO) analysis of QR-regulated DEGs was performed using the Coexpedia platform (https://www.coexpedia.org/).

### 2.2 Network pharmacology analysis

#### 2.2.1 Collection of QR targets and representative indication targets

The potential indications enriched in DO combined with the pharmacological effects of approved drugs screened by CMap were used for drug repurposing of QR, and the most representative indication was selected for subsequent research. The representative indication targets were retrieved based on MalaCards (https://www.malacards.org/), TTD, PharmgKB (https://www.pharmgkb.org/), OMIM and DisGeNET (https://www.disgenet.org/). In addition, to collect the QR targets, CTD (https://ctdbase.com), DrugBank (https://go.drugbank.com/), PubChem (https://pubchem.ncbi.nlm.nih.gov/), HIT (http://www.badd-cao.net:2345/), PharmMapper (http://www.lilab-ecust.cn/pharmmapper/), and SwissTargetPrediction (http://www.swisstargetprediction.ch/) databases were used. The QR targets were matched with the targets of the relevant indication to obtain the common targets of QR treatment indication.

#### 2.2.2 Protein-protein interaction (PPI) network construction

The common targets were put into the STRING database (https://cn.string-db.org/), where the organism was set as “*homo sapiens*” and the data file was imported into Cytoscape for interaction network visualization. Then, the CytoHubba plugin (http://apps.cytoscape.org/apps/cytohubba) was 103 used to analyze the PPI network and calculate the core targes.

#### 2.2.3 Functional enrichment analysis

All the common targets were imported into Metascape (https://metascape.org/) for functional enrichment analysis (Duan et al., 2019; Duan et al., 2021). The Gene Ontology (GO) and Kyoto Encyclopedia of Genes and Genomes (KEGG) enrichment results with significant statistical differences (*p* < 0.05) were visualized using the OmicShare tools (https://www.omicshare.com/tools).

### 2.3 Animal experimental verification

#### 2.3.1 Animals management and drug preparation

C57BL/6 female mice, 8–10 weeks of age (18–20 g), were obtained from SPF Biotechnology Co., Ltd (Beijing, China), and housed in specific pathogen-free conditions at the First Affiliated Hospital of Henan University of Chinese Medicine (animal ethics committee approval No. YFYDW2023025), Henan, China. All efforts were made to reduce animal suffering, and the Institutional Committee on Care and Use of Research animals approved the procedures used in this study. The inactivated *Mycobacterium tuberculosis* H37Ra (No. 231141, Difco) was added to Freund’s incomplete adjuvant (No. F5506, Sigma) to prepare the 10 mg ml^-1^ solution, and the same volume of myelin oligodendrocyte glycoprotein peptide (MOG35-55, MEVGWYRSPFS-RVVHLYRNGK; L2880; GenScript) solution (2 mg ml^-1^) was dissolved in sterile PBS to prepare oil antigen emulsion.

#### 2.3.2 Experimental autoimmune encephalomyelitis (EAE) model induction and animal behavior test

The C57BL/6 mice were randomly divided into the normal control (NC) group, the EAE group and EAE + QR group (n = 6 in each group). EAE was induced by MOG 35-55, as previously described (M. L. Zhang et al., 2017). Briefly, each mouse was anesthetized with 1% phenobarbital sodium and then immunized subcutaneously. On days 0 and 2 post-immunization (p.i.), 200 ng pertussis toxin (No. 180, List Biological) was intraperitoneally injected into mice. The treatment by oral gavage started from day 10 p.i. The EAE + QR group mice were treated with 50 mg (kgd)^−1^ QR (Hendriks et al., 2004) (No. C10798568, Macklin) suspension prepared by using 0.5% sodium carboxymethyl cellulose (CMC-Na) as the vehicle. NC and EAE groups mice were treated with 0.5% CMC-Na in equal volumes. The EAE model was scored on a five-point scale for neurological function (Glenn et al., 2017): 1-animal tail weakness; 2-weakness of hind limbs; 3-hind limb paralysis; 4-anterior and posterior limbs were paralyzed; 5-dying or dying. In addition, NC group mice were used as naive control. The above trials were repeated twice to ensure the reproducibility of clinical efficacy.

#### 2.3.3 Hematoxylin-Eosin (HE) and Luxol Fast Blue (LFB) stainings

To observe mice’s central nervous system (CNS) inflammation and demyelination. The mice were anesthetized with 1% pentobarbital sodium and perfused with 0.9% sodium chloride solution on day 21 p.i. The left brain was quickly removed and fixed with 4% paraformaldehyde, embedded in paraffin, sectioned, and stained with HE inflammatory infiltration staining and LFB myelin staining. The scoring criteria for inflammatory infiltration (Zhao et al., 2018) were as follows: 0-no sign of inflammation; 1-scattered inflammatory cells; 2-some inflammatory cells and karyopyknosis; 3-perivascular inflammatory cell infiltrate; 4-marked inflammatory cell infiltration into the parenchyma. Demyelination level scoring criteria (Dou et al., 2021) were as follows: 0-no demyelination; 1-rare demyelination; 2-a few demyelinated areas; 3-a large area of demyelination.

### 2.4 Molecular docking

To analyze the binding affinities and modes of interaction between QR and the core target proteins, AutoDock Vina, a silico protein-ligand docking software, was employed (Eberhardt et al., 2021). The two-dimensional structure of QR was retrieved from the PubChem database and then converted to MOL2 format using ChemDraw software. The main target protein was also retrieved from the Protein Data Bank (https://www.rcsb.org/). For docking analysis, all protein and molecular files were converted into PDBQT format with all water molecules excluded, and polar hydrogen atoms were added. The molecular docking results were evaluated through the criteria of binding structure, binding energy, and possible interactions between the ligand and the critical residues of the protein.

### 2.5 Molecular dynamics simulation

After molecular docking, molecular dynamics simulation was used to simulate the complex of target proteins and QR to detect the stability and flexibility of the complex. This study used the GROMACS 2020.5 software package, GROMOS54a7_atbff force field, and SPC216 water model for molecular dynamics simulation. In order to ensure the total charge neutrality of the simulation system, a corresponding number of sodium ions are added to the system to replace water molecules to form a solvent box of appropriate size. Then the periodic boundary condition is applied to the three directions of the system. Use the gromos54a7_atb force field to obtain the QR’s force field parameters from ATB (http://atb.uq.edu.au/) database. Initially, the entire system used the steepest descent minimization. Then, the balance of acceptor, ligand, and solvent was achieved by running 1 ns NVT. Finally, molecular dynamics sampling with 100 ns and a time step of 2 fs was performed for analysis.

### 2.6 Reverse transcription-polymerase chain reaction (RT-PCR)

Total RNA was extracted from mouse spinal cord tissues by Tissue RNA Purification Kit (ESscience Biotech, China) and reversely transcribed into cDNA using Fast All-in-One RT Kit (ESscience Biotech, China). Then, RT-PCR was performed using cDNA as the template with Super SYBR Green qPCR Master Mix (ESscience Biotech, China) and QuantStudio 6 Flex PCR System (Applied Biosystems, United States). GAPDH was used as the reference control, and the results were analyzed using the 2^−ΔΔCT^ method. The primer sequences of target genes are shown in Table 1.

**TABLE 1:** Primer sequences of target genes.

Gene	Forward sequence (5’–3′)	Reverse sequence (5’–3′)
TNF-α	GAC​GTG​GAA​CTG​GCA​GAA​GAG	TTG​GTG​GTT​TGT​GAG​TGT​GAG
IL-6	CCA​AGA​GGT​GAG​TGC​TTC​CC	CTG​TTG​TTC​AGA​CTC​TCT​CCC​T
IL-1β	GCA​ACT​GTT​CCT​GAA​CTC​AAC​T	ATC​TTT​TGG​GGT​CCG​TCA​ACT
IFN-γ	ATG​AAC​GCT​ACA​CAC​TGC​ATC	CCA​TCC​TTT​TGC​CAG​TTC​CTC
IL-17A	TTT​AAC​TCC​CTT​GGC​GCA​AAA	CTT​TCC​CTC​CGC​ATT​GAC​AC
IL-2	TGA​GCA​GGA​TGG​AGA​ATT​ACA​GG	GTC​CAA​GTT​CAT​CTT​CTA​GGA​CA
GAPDH	AGG​TCG​GTG​TGA​ACG​GAT​TTG	TGT​AGA​CCA​TGT​AGT​TGA​GGT​CA

### 2.7 Statistical analysis

All statistical analyses were performed using GraphPad Software (GraphPad Prism 9, United States). The Student’s t-test was used to analyze the weight changes, the difference in the mean clinical severity of mice, and HE staining and LFB staining scores. All values are presented as mean ± standard deviation (*SD*). *p* < 0.05 was considered statistically significant.

## 3 Results

### 3.1 Drug repurposing analysis

#### 3.1.1 QR-regulated DEGs on human monocytes

**FIGURE 2:**
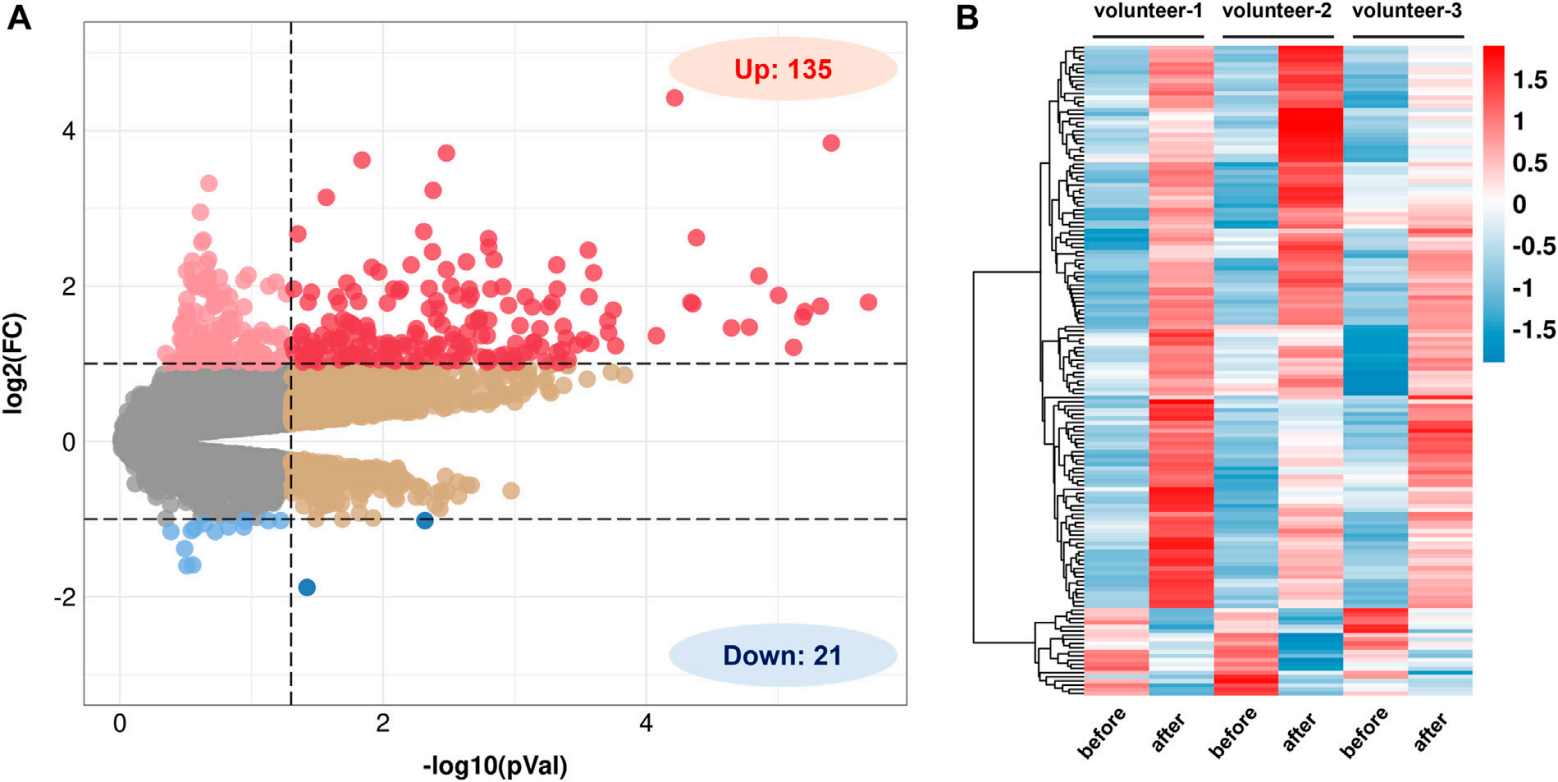
Screening of differentially expressed genes (DEGs) regulated by QR. **(A)** Volcano diagram of QR-regulated DEGs in GSE13899 (*p* < 0.05 & |log *FC*| > 1). **(B)** Heat map of QR-regulated DEGs. Red represents upregulated genes, and blue represent downregulated genes.

Based on the GEO database, a gene expression dataset numbered GSE13899 was screened (Boomgaarden et al., 2010). The dataset contains gene expression profiles of CD14^+^ monocytes before and after 2 weeks of QR (150 mg d^-1^) supplementation in three healthy volunteers. The results show that a total of 156 DEGs of QR have been screened, including 135 upregulated DEGs and 21 downregulated DEGs with *p*-value <0.05 and |log *FC*| > 1 as the threshold: (Figure 2).

#### 3.1.2 Approved drugs query and DO enrichment analysis

**FIGURE 3:**
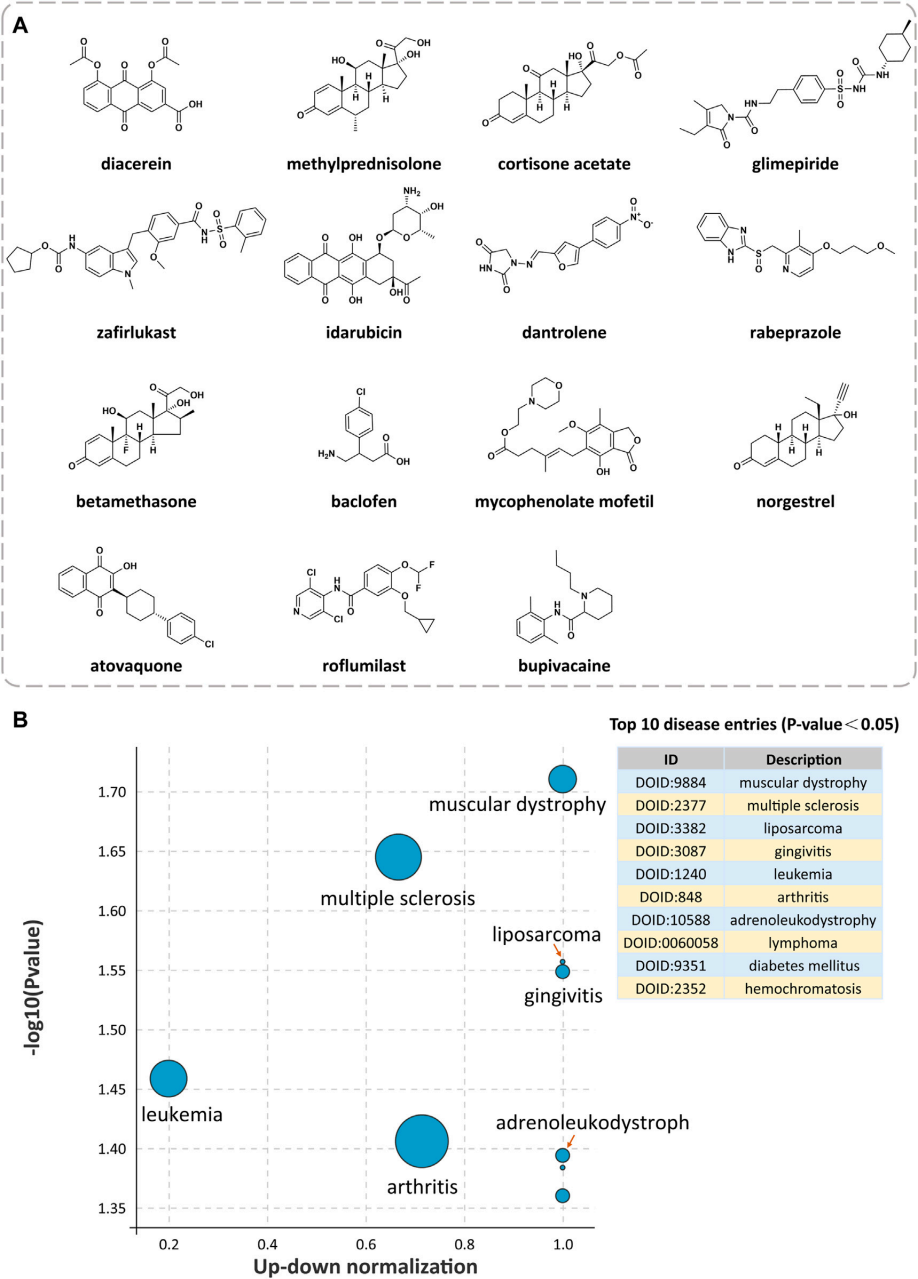
Exploration of potential indications for drug repurposing of QR. **(A)** The chemical structures of the top 15 approved drugs are similar to QR gene expression signatures. **(B)** Disease Ontology (DO) enrichment analysis bubble diagram of QR-regulated DEGs.

Under the condition of Norm CS ≥ 1.3, we screened 2312 compounds with similarity to QR gene expression signatures. According to the DrugBank database, the top 15 approved drugs of the above compounds were screened for the potential pharmacological effects and clinical indications of QR (Table 2; Figure 3A). The results revealed that the clinical pharmacological effects of the top 15 approved drugs are mainly involved in immune, nervous, endocrine, respiratory, digestive, and other systems, including anti-inflammatory, immunosuppressive, hypoglycemic, anti-tumor, anti-asthma, antimalarial, suggesting that QR may have the similar pharmacological effects and clinical indications.

**TABLE 2:** Top 15 approved drugs with high similarity to the Quercetin (QR) gene expression signatures.

Candidate name	Norm CS	Drug category	Indication	Pharmacological action
diacerein	1.67	anti-inflammatory agents	Osteoarthritis Almezgagi et al. (2020)	interleukin inhibitor; anti-inflammation
methylprednisolone	1.47	corticosteroids	allergic and autoimmune inflammatory diseases [including multiple sclerosis (**MS** ^*^)] Wang et al. (2020b)	glucocorticoid receptor agonist; anti-inflammation and immunosuppression
cortisone acetate	1.46	corticosteroids	adrenal cortical hypofunctions and **MS** ^**#**^ Andreini et al. (2002)	glucocorticoid receptor agonist; anti-inflammation and immunosuppression
glimepiride	1.40	hypoglycemic agents	type 2 diabetes mellitus Gudipaty et al. (2014)	insulin secretagogue; decrease blood glucose
zafirlukast	1.38	anti-asthmatic agents	asthma and **MS** ^**#**^ Wang et al. (2011)	leukotriene receptor antagonist; reduce tracheal contraction and inflammation
idarubicin	1.37	antineoplastic and immunomodulating agents	acute leukemia Kadia et al. (2021)	topoisomerase inhibitor
dantrolene	1.34	muscle relaxants	malignant hyperthermia Rana et al. (2022); skeletal muscle spasm caused by **MS** ^*^ Fragoso et al. (2020)	calcium channel blocker; inhibition of sarcoplasmic reticulum release of calcium to reduce muscle contraction
rabeprazole	1.33	anti-ulcer agents	acid-reflux disorders and peptic ulcer disease	proton pump inhibitor; gastric acid secretion inhibition
betamethasone	1.32	corticosteroids	allergic and autoimmune inflammatory diseases (including **MS** ^*^) Rana et al. (2022)	glucocorticoid receptor agonist; anti-inflammation and immunosuppression
baclofen	1.31	muscle relaxants	skeletal muscle spasm caused by **MS** ^*^ Fragoso et al. (2020)	GABA receptor agonist; skeletal muscle relaxant; anti-inflammatory and neuroprotective activities
mycophenolate mofetil	1.31	immunosuppressive agents	transplanted organ rejection and **MS** ^*^ Pimentel Maldonado et al. (2023)	immunosuppressant; blocking the proliferation of T and B cells
norgestrel	1.31	progestin contraceptives	abnormal uterine bleeding and endometriosis	progesterone receptor agonist; contraception
atovaquone	1.30	antimalarials	plasmodium falciparum malaria	mitochondrial inhibitor; antimalarial
roflumilast	1.30	phosphodiesterase inhibitors	exacerbation of chronic obstructive pulmonary disease and psoriasis vulgaris	phosphodiesterase inhibitor; increased levels of intracellular cAMP
bupivacaine	1.30	central nervous system depressants	a wide variety of superficial and invasive procedures	sodium channel inhibitor; blocking nerve excitation and conduction

**Norm CS:** normalized connection score; **MS** ^*^ **:** confirmed by clinical trials; **MS** ^**#**^ **:** confirmed by animal experiments.

Based on Coexpedia platform, the QR-regulated DEGs are mainly enriched in 84 significant disease entries (*p* < 0.05), and the top 10 disease entries were screened for display according to the *p*-value. As shown in Figure 3B, the primary potential indications for QR include neuromuscular diseases (muscular dystrophy), autoimmune diseases (multiple sclerosis), mouth diseases (gingivitis), joint diseases (arthritis), neoplasms (liposarcoma, leukemia, and lymphoma), metabolic diseases (diabetes mellitus and hemochromatosis), and CNS diseases (adrenoleukodystrophy). Integrating analysis of the above clinical indications cured by the top 15 approved drugs and the top 10 enriched DO diseases, the result found that the potential indications of QR are primarily related to multiple sclerosis (MS), osteoarthritis, type 2 diabetes mellitus, and acute leukemia. Notably, 7 of the above 15 top drugs have been confirmed to have better therapeutic effects on MS, including five drugs already used clinically and two drugs proven by animal experiments. Therefore, MS was selected as a representative disease for the subsequent study to verify the feasibility of the drug repurposing method for QR.

### 3.2 Network pharmacology

#### 3.2.1 PPI network analysis of potential targets of QR in the treatment of MS

**FIGURE 4:**
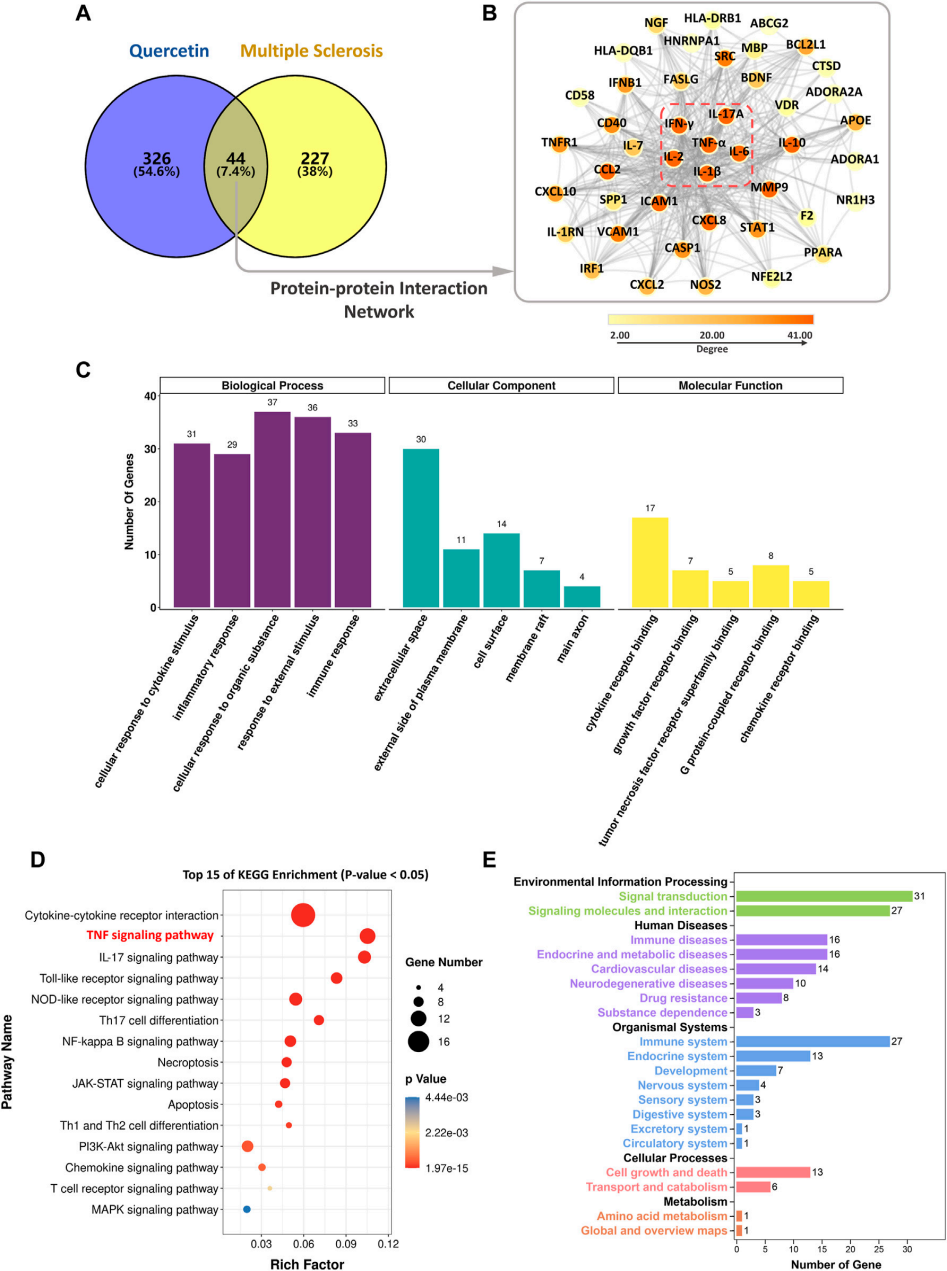
The potential mechanism of QR in the treatment of MS. **(A)** Venn diagram of common targets. **(B)** The protein-protein interaction network (red dotted line as the core targets) **(C)** Gene Ontology enrichment analysis results. **(D)** Kyoto Encyclopedia of Genes and Genomes (KEGG) pathway enrichment analysis results. **(E)** KEGG functional annotation results.

After identifying MS as a potential indication for QR therapy, targets were collected in the disease database using the keywords “multiple sclerosis”, “relapsing-remitting multiple sclerosis”, “secondary progressive multiple sclerosis”, “primary progressive multiple sclerosis”, and “progressive recurrent multiple sclerosis” respectively. As shown in the Venn diagram (Figure 4A), 370 QR and 271 MS targets were collected, with 44 common targets. Through the analysis of the PPI network (Figure 4B) by CytoHubba, six core targets were screened out, which were tumor necrosis factor (TNF-α), interleukin 6 (IL-6), interleukin 1 β (IL-1β), interferon γ (IFN-γ), interleukin 17A (IL-17A), and interleukin 2 (IL-2), respectively. These targets are closely related to the inflammatory immune response of autoimmune diseases (Ge et al., 2018), suggesting that QR may treat MS mainly by affecting these targets.

#### 3.2.2 Functional enrichment analysis

The results of the Gene Ontology (GO) enrichment analysis are shown in Figure 4C. As for the biological process, QR affected cellular response to cytokine stimulus, inflammatory response, cellular response to an organic substance, response to external stimulus, and immune response. For the cellular component, QR has mainly located in the exterior of the cell plasma membrane, membrane, and axon. For the molecular function, the effects of QR on MS were related to the binding of a cytokine receptor, growth factor receptor, TNF receptor superfamily, G protein-coupled receptor, and chemokine receptor.

The KEGG enrichment indicated that 44 common targets were enriched in 87 pathways (*p* < 0.05). The top 15 main pathways are TNF signaling pathway, IL-17 signaling pathway, Toll-like receptor signaling pathway, NOD-like receptor signaling pathway, and NF-κB signaling pathway (Figure 4D). KEGG functional annotation (Figure 4E) indicates that QR may mainly affect the immune system by regulating cell signaling pathways, thereby interfering with immune and neurodegenerative diseases, such as MS.

### 3.3 Experimental verification

#### 3.3.1 The therapeutic effects of QR on EAE mice

**FIGURE 5:**
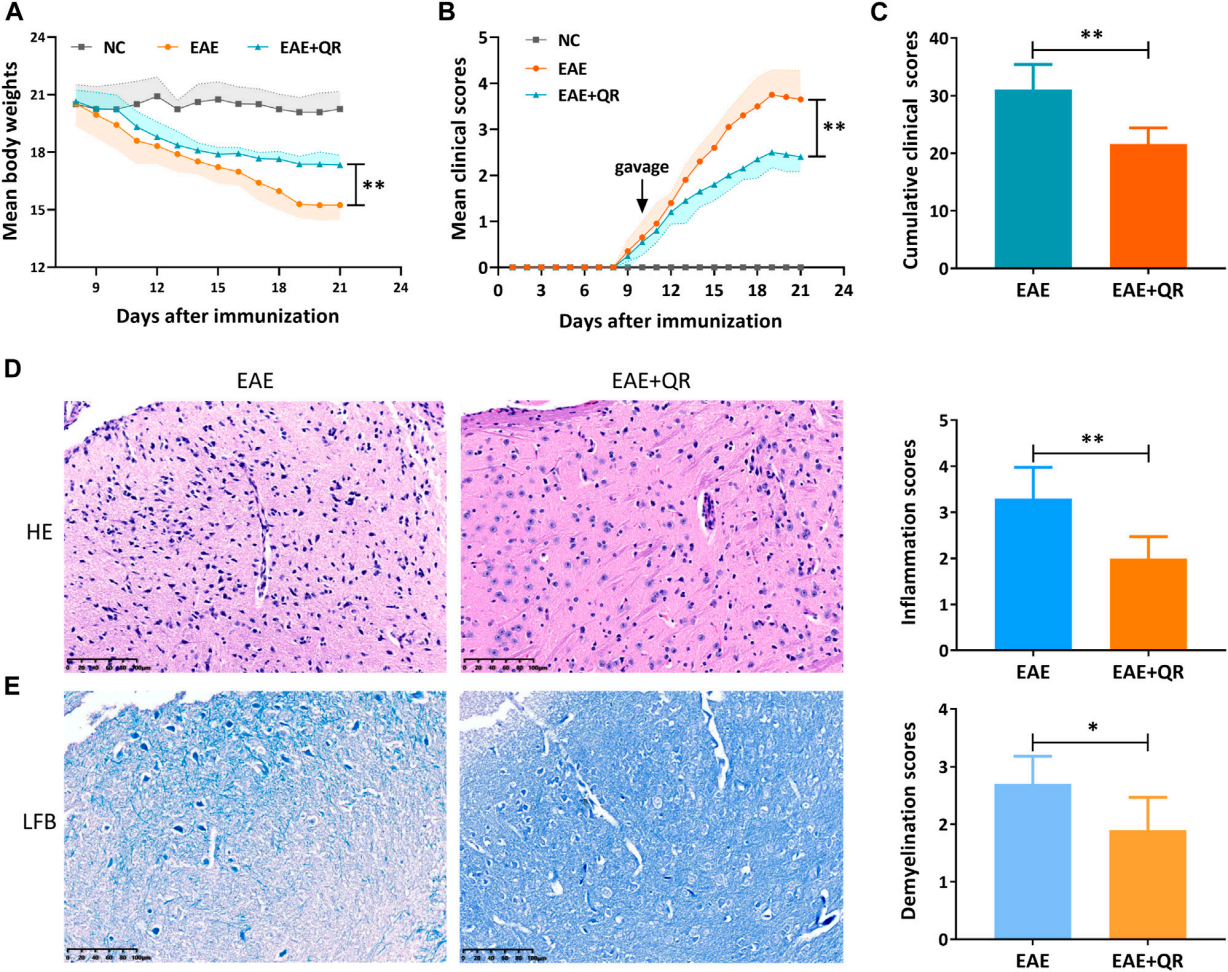
QR treatment ameliorated EAE severity, inflammatory infiltration, and demyelination. **(A)** mean body weights. **(B)** mean clinical scores. **(C)** cumulative clinical scores. **(D)** Hematoxylin-Eosin (HE) staining and inflammation scores. **(E)** Luxol Fast Blue (LFB) staining and demyelination scores. Data are shown as mean ± *SD* (n = 6). ^*^ *p* < 0.05; ^**^ *p* < 0.01, compared to EAE group. Scale bar, 100 μm.

The symptoms such as loss of tail tonicity, staggering gait, hind-limb paralysis, four-limb paralysis, and even death appeared sequentially in EAE mice. The first clinical score of the CMC-Na-treated EAE group mice appeared around day 9 p.i. and increased in the following days. The disease peaked on day 19 p.i., with reduced body weight (Figure 5A). Notably, the EAE + QR group mice were heavier (*p* < 0.01), and the mean clinical scores and cumulative clinical scores were lower (*p* < 0.01) than the EAE group mice (Figures 5B, C).

All mice were sacrificed on day 21 p.i., and the brain was collected for pathological analysis. HE staining and LFB staining of the brain showed an extensive infiltration of inflammatory cells, perivascular cuffing and large areas of demyelination in the CMC-Na-treated EAE group, while QR treatment significantly inhibited the infiltration of inflammatory cells into the CNS of EAE mice (*p* < 0.01, Figure 5D), with decreased demyelination (*p* < 0.05, Figure 5E). These results indicated that QR had the potential to ameliorate EAE.

#### 3.3.2 Molecule docking

TNF-α was the most connected target (degree = 41) to other PPI network protein nodes. Studies have shown that TNF-α binding to Tumor Necrosis Factor Receptor Type 1 (TNFR1) can trigger a series of proinflammatory cytokines and chemokines, thereby increasing the immune cascade of CNS (Hilliard et al., 2020; Taghipour et al., 2022). In addition, combined with the results of drug repurposing of QR, we speculate that QR may play an anti-inflammatory and immunosuppressive pharmacological role by inhibiting TNF-α and TNFR1, thereby alleviating the autoimmune response of MS. Therefore, this study used QR as a docking ligand, TNF-α (PDB ID, 2AZ5; resolution, 2.1 Å) and TNFR1 (PDB ID, 1FT4; resolution, 2.9 Å) as docking target proteins. The reported small molecule inhibitors of these two target proteins were downloaded from the PDB database, and the active sites of the inhibitors were used as the docking pockets of QR. Before QR docking with these two protein targets, all their small molecule inhibitors should be extracted.

**FIGURE 6:**
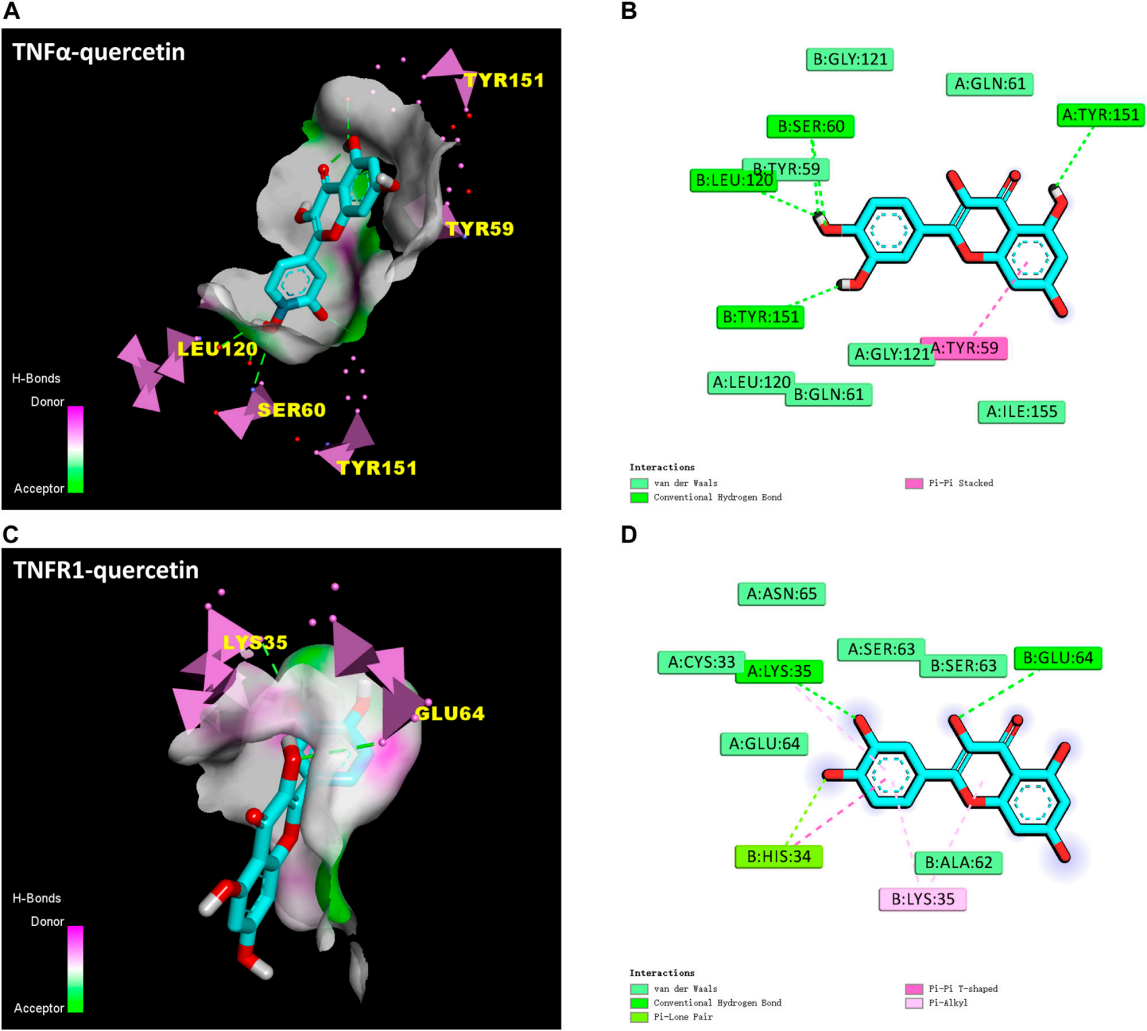
Molecular docking results of QR with TNF-α (PDB ID: 2AZ5) and TNFR1 (PDB ID: 1FT4). **(A)** The hydrogen bond surface of TNFα-QR. **(B)** Interactions between QR and amino acid residues of TNF-α protein. **(C)** The hydrogen bond surface of TNFR1-QR. **(D)** Interactions between QR and amino acid residues of TNFR1 protein.

The docking protocol was validated by redocking the co-crystallized inhibitors “307” (He et al., 2005) and “703” (Carter et al., 2001) of these two proteins to their active sites, respectively. Root mean square deviation (RMSD) between the docked structures and the initial structures was less than 2.00 Å. The redocking results of “307” and “703” signified that Auto Dock Vina software is reliable (Trott and Olson, 2010). The protocol can be used for docking other compounds. It is generally believed that the binding energy < −4.25 kcal mol^-1^ indicates that there is a certain binding activity between the ligand small molecule and the receptor protein; the binding energy < −5.0 kcal mol^-1^ indicates that there is a good binding activity between the two; the binding energy < −7.0 kcal mol^-1^ indicates that the ligand and receptor have strong binding activity (Hsin et al., 2013). Reassuringly, results showed that QR bound to TNF-α and TNFR1 protein targets through visible hydrogen bonds and strong electrostatic interactions. Moreover, the original inhibitor active sites of these two target proteins were successfully docked by QR, and had low binding energy of −7.1 (2AZ5) and −6.7 (1FT4) kcal·mol^-1^, indicating highly stable binding (Table 3; Figure 6). This suggested that QR might play an anti-inflammatory and immunosuppressive effects by inhibiting TNF-α and TNFR1 targets.

**TABLE 3:** Docking scores of QR with TNF-α and TNFR1.

Compound	Target proteins	Binding scores (kcal mol^-1^)	PDB ID	Coordinates of docking-pocket	Combining with the amino acid residues
Quercetin	TNF-α	−7.1	2AZ5	*x* (−19.163), *y* (74.452), *z* (33.837)	TYR-51, GLN-61, GLY-121, SER-60, TYR-59, LEU-120, TYR-151, ILE-155
	TNFR1	−6.7	1FT4	*x* (19.583), *y* (−2.944), *z* (24.173)	GLU-64, SER-63, ASN-65, CYS-33, LYS-35, HIS-34, ALA-62

#### 3.3.3 Molecular dynamics simulation

**FIGURE 7:**
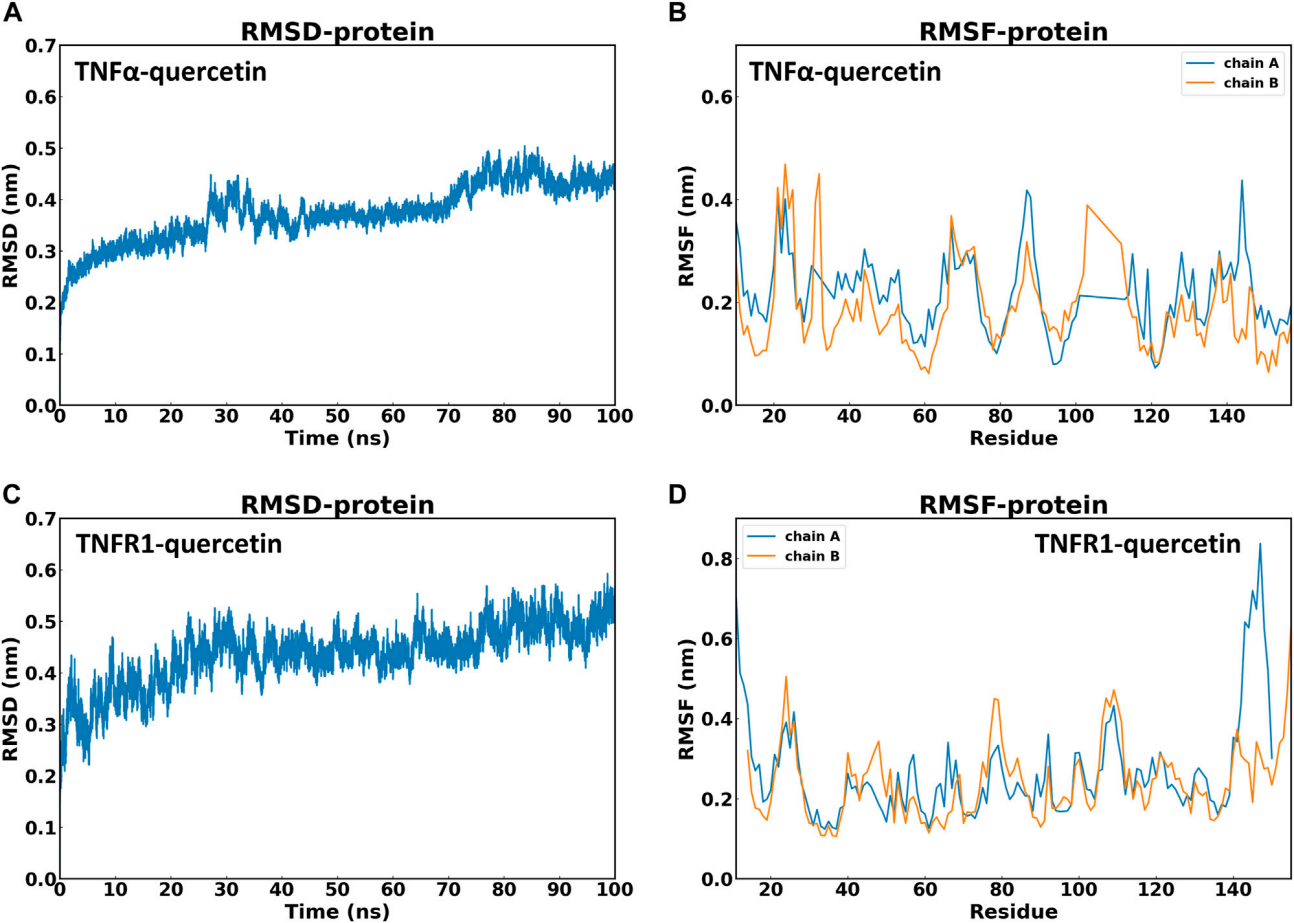
Stability of QR with TNF-α and TNFR1 in 100 ns molecular dynamics simulation. **(A)** Root mean square deviation (RMSD) values of TNFα-QR protein complex. **(B)** Root Mean Square Fluctuation (RMSF) values of amino acids of protein TNF-α. **(C)** RMSD values of the TNFR1-QR protein complex. **(D)** RMSF values of amino acids of protein TNFR1.

Based on molecular dynamics simulation, the data of RMSD and Root Mean Square Fluctuation (RMSF) of TNFα-QR and TNFR1-QR in protein were obtained. Compared with the first frame, the TNFα-QR complex reached stability after 10 ns(RMSD value is 0.30–0.44 nm). The RMSD oscillation amplitude of QR is about 0.14 nm TNFR1-QR complex was stable after 28 ns(RMSD 0.46–0.56 nm). The RMSD oscillation amplitude of QR is about 0.10 nm. It is suggested that during the 100 ns simulation process, the systems of TNFα-QR and TNFR1-QR quickly reached stability without excessive fluctuations (Figures 7A, C).

Another stability factor is the fluctuations of protein during simulation that were evaluated by analyzing RMSF values (Figures 7B, D). On RMSF values, TNF-α and TNFR1 active site residues interacting with QR were all stable with RMSF values below 0.2nm. In particular, most of the RMSF values of the residues that form hydrogen bonds between TNFα-QR (TYR151, SER60, TYR59, and LEU120) and TNFR1-QR (LYS35 and GLU64) complexes are less than 0.15 nm. It can be inferred that QR fits nicely on the active site and maintains interaction with amino acid residues.

#### 3.3.4 Validation of potential core targets of QR-treated EAE mice

**FIGURE 8:**
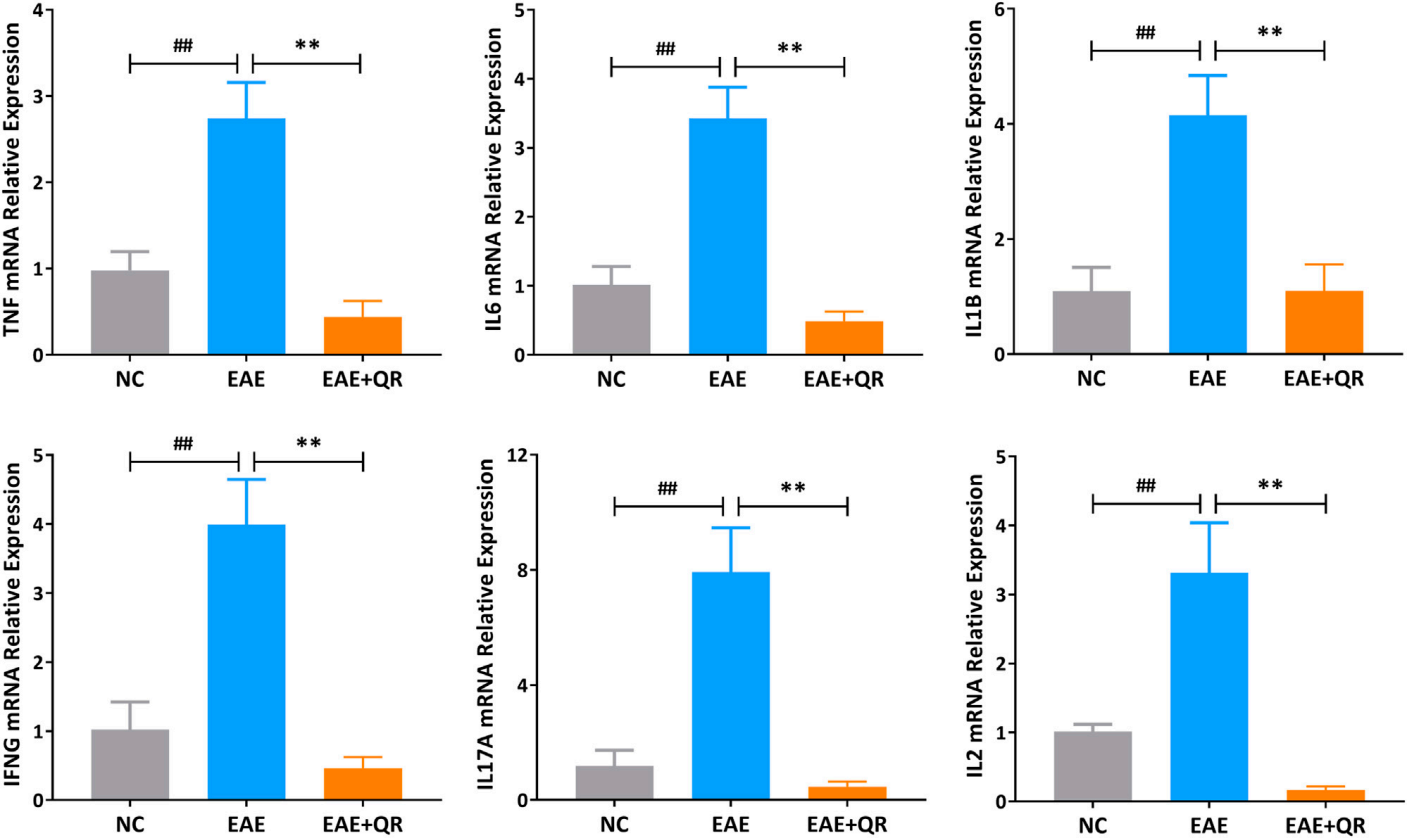
The mRNA levels of 6 core target genes in the spinal cord tissues of mice in NC, EAE, and EAE + QR groups were detected by RT-PCR. Data are shown as mean ± *SD* (n = 6). ^##^ *p* < 0.01, compared to NC group; ^**^ *p* < 0.01, compared to the EAE group.

Six potential core target genes (TNF-α, IL-6, IL-1β, IFN-γ, IL-17A, and IL-2) were identified by PPI network analysis. To verify the above findings, we determined the mRNA levels of these six target genes in the spinal cord tissues of NC, EAE, and EAE + QR groups by reverse transcription-polymerase chain reaction (RT-PCR). The expressions of TNF, IL6, IL1B, IFNG, IL17A, and IL2 in the CNS of EAE mice were elevated (all *p* < 0.01) observably compared with that in NC mice, while their expressions were significantly reduced (all *p* < 0.01) after QR treatment (Figure 8).

## 4 Discussion

Based on the drug repurposing strategy, this study speculated that QR with various pharmacological activities might potentially treat MS, osteoarthritis, type 2 diabetes mellitus, and acute leukemia through gene expression profile combined with CMap.

Among the drugs screened by CMap, corticosteroids (methylprednisolone, cortisone acetate, betamethasone) mainly treat allergic and autoimmune inflammatory diseases (including MS) through anti-inflammatory and immunosuppressive effects (Fischer et al., 2019). Muscle relaxants (dantrolene, baclofen) can inhibit the excitement of the nervous system and reduce calcium influx to alleviate skeletal muscle spasms caused by MS (Fragoso et al., 2020). Anti-inflammatory agents (diacerein) inhibit the inflammatory response of osteoarthritis by reducing the level of interleukin-1β activity (Fidelix et al., 2014). Hypoglycemic agents (glimepiride) lower blood sugar by stimulating pancreatic β cells to release insulin (Halvorsen et al., 2023). Antineoplastic agents (idarubicin) treat acute leukemia by inhibiting topoisomerase II and reducing nucleic acid synthesis (Mao et al., 2022). The pharmacological effects and indications of the above drugs suggest that QR may have anti-inflammatory, hypoglycemic, anti-tumor, anti-spasm, immunosuppressive, and other potential pharmacological effects. Interestingly, anti-inflammatory and hypoglycemic are known biological activities of QR (Yang et al., 2020; Yi et al., 2021), indicating that it is reliable to discover the potential pharmacological effects of QR through drug repurposing strategy. It is worth noting that, as shown in Table 2; Figure 3B, seven approved drugs were found to be related to the treatment of MS, and MS ranked high in DO enrichment analysis. Therefore, we conducted a follow-up study with MS as a representative indication of QR.

MS is an autoimmune disease of the CNS characterized by immune cell infiltration and demyelination. It has the characteristics of easy recurrence and a high disability rate. This study used an internationally recognized animal model of MS (EAE) to verify whether QR has a therapeutic effect on MS (Oh et al., 2018). The results showed that after drug intervention, QR indeed delayed the pathogenesis of EAE mice, weakened the degree of disease, and alleviated the pathological changes such as inflammatory infiltration and demyelination of the CNS, suggesting that it is feasible to find new indications of QR based on drug repurposing strategy.

It is currently recognized that the pathogenesis of MS is caused by the cellular immune response mediated by the body’s own T lymphocytes (Reich et al., 2018). Notably, based on the PPI network, this study screened out six inflammatory cytokines secreted by activated T lymphocytes, such as IL-2, IL-17A, IL-1β, IL-6, IFN-γ, TNF-α. Studies have shown that IL-2 is an immune-stimulating factor required for T lymphocyte expansion, and activated T lymphocytes can further secrete various inflammatory factors to aggravate the CNS immune response (Pol et al., 2020). IL-17A secreted by T-helper 17 cells can destroy the blood-brain barrier and activate astrocytes and microglia, thereby triggering the inflammatory cascade of the CNS (Waisman et al., 2015). IL-1β produced by microglia in the CNS helps to expand the number of microglia in an autocrine manner and increases the production of inflammatory cytokines and chemokines, thereby promoting the pathogenesis of MS (C. J. Zhang et al., 2018; Zhai et al., 2021). IL-6 is continuously expressed in astrocytes in the demyelinated area of EAE mice. In addition, low expression of IL-6 inhibits the proliferation of inflammatory cells and alleviates the inflammatory response of the CNS, suggesting that IL-6 may play an essential role in the progression of MS (Savarin et al., 2015). IFN-γ stimulates microglial polarization toward proinflammatory type 1 (M1), thereby aggravating CNS axonal injury and demyelination (Orihuela et al., 2016; Maurya et al., 2021). The binding of TNF-α to TNFR1 triggers a series of proinflammatory cytokines and chemokines to aggravate the immune response in MS (Hilliard et al., 2020; Skartsis et al., 2022). In the EAE animal model, it was found that TNF-α can induce oligodendrocyte necrosis and aggravate the loss of myelin sheath, while TNF-α inhibitors can prevent neurological dysfunction in EAE mice (Martin et al., 1995; Ofengeim et al., 2015). The EAE model was constructed based on chimeric human/mouse TNFR1 knock-in mice, and it was found that the course of TNFR1 deficiency mice was significantly improved (Williams et al., 2018). The molecular docking and molecular dynamics simulation results showed that QR had a stable binding effect with the inhibitor-active sites in TNF-α and TNFR1 target proteins. QR may exert anti-inflammatory and immunosuppressive effects by blocking TNF-α/TNFR1 signaling and reducing the immune response of CNS. Indeed, the above inflammatory cytokines were significantly higher in the CNS, cerebrospinal fluid, and blood of MS patients than in healthy volunteers (Magliozzi et al., 2018; Kunkl et al., 2020). Gratifyingly, this study found that QR can significantly downregulate the gene expression of IL-2, IL-17A, IL-1β, IL-6, IFN-γ, and TNF-α. In addition, according to several studies, in the protein detection results of ELISA and Western blot, QR, which has a strong anti-inflammatory effect, can significantly inhibit the protein expression of inflammatory cytokines IL-2, IL-17A, IL-1β, IL-6, IFN-γ and TNF-α (Meng et al., 2018; Li et al., 2019; Chen et al., 2022). In summary, QR may inhibit the immune response of CNS by regulating the expression of these inflammatory cytokines, thereby reducing the incidence of MS.

In conclusion, based on the drug repurposing strategy, we have successfully discovered new clinical indications of QR and verified QR’s therapeutic potential and potential mechanism on MS through experiments. This study laid an experimental foundation for the preclinical study of QR in treating MS and provided a reference for expanding the clinical indications of other approved or investigational drugs. Of course, as an early exploratory study, a series of more in-depth studies on the mechanism of action are still needed. Thus, this study must be viewed in light of its strengths and limitations, many of which represent opportunities for future research.

## Data Availability

The original contributions presented in the study are included in the article/Supplementary material, further inquiries can be directed to the corresponding authors.
